# The Position of the Voluntary Hospitals in the National Health Services

**Published:** 1936

**Authors:** Robert F. Barclay


### THE POSITION OF THE VOLUNTARY HOSPITALS IN THE NATIONAL HEALTH SERVICES.

---

A DEPARTMENTAL COMMITTEE has recently been inquiring into the Scottish Health Services with a view to their extension and co-ordination. It is understood that some extension of the scope of the National Health Insurance Act is contemplated, and that Hospital and Specialist Services may be included in the sphere of operation of the Act.

The Voluntary Hospitals in Glasgow and the West of Scotland submitted a Memorandum and gave evidence before the Committee. By the courtesy of Dr. Robert F. Barclay this *Journal* is permitted to publish a Synopsis prepared by him of the Memorandum. Although the conditions in the West of Scotland are not identical with those in the West of England, the admirable manner in which the case for the Voluntary Hospitals is summed up in Dr. Robert F. Barclay's synopsis will be appreciated by the members of our Society and readers of the *Journal.*

### A SYNOPSIS

prepared by ROBERT F. BARCLAY, LL.D., Chairman of the Royal Hospital for Sick Children, Glasgow, of the Memorandum presented by Eighteen Voluntary Hospitals in Glasgow and the West of Scotland (hereinafter referred to as the said Voluntary Hospitals).

*1st November*, 1935.

1. Voluntary Hospitals are well worth preserving for many reasons, *e.g.* :—

(*a*) They have a very real place in the hearts of the public, as the subscription lists show.

(*b*) The large number who require hospital treatment appreciate the services of the Voluntary Hospitals very highly, and take considerable trouble, and are even prepared to wait, to get admission to such Hospitals.

(*c*) The Boards of Voluntary Hospitals are notable for the number of business men of standing and undoubted capacity who give their services freely to promote the usefulness and secure the good management of these institutions.

(*d*) If it were not for the large sum provided each year voluntarily for the maintenance of the Voluntary Hospitals a much larger sum would require to be raised by increased taxation.

(*e*) They have done invaluable work in the past, and are capable of doing much good work in the future.

2. The said Voluntary Hospitals see their way to continue and to increase their usefulness as they have done in the past provided :—

(*a*) They are not subjected to unfair or undue competition on the part of Local Authorities and rate-aided hospitals having the public purse at their back.

(*b*) The important, satisfactory and equitable provisions of Section 27 of the Local Government Act for Scotland, 1929, are adhered to by the Department of Health and loyally observed by Local Authorities.

(*c*) That they are recognized by the State as an integral part of the health services of the country, and that the fear which has somehow evolved in recent years of drastic changes involving in some cases practical extinction of some, if not many, Voluntary Hospitals, is removed and confidence restored.

3. The Committee will doubtless realize that there is an immense difference in the attitude of the various Local

Authorities, urban and rural, to Voluntary Hospitals. In some cases there is satisfactory co-operation ; in other cases it would appear as if the ultimate aim is to supersede Voluntary Hospitals altogether, although doubtless this would at present not be admitted.

4. Voluntary Hospitals have accomplished in the past and are undertaking now a work of great national importance. They have to a very material extent met the needs of the country, and they propose and desire to continue to do so. They have always recognized, however, that they cannot meet all the hospital services required by the community. Indeed, they have not regarded it as their province to do so. Their efforts have always been supplemented by services provided by the Local Authorities. The word co-operation is much used in this connection, and rightly so, but co-operation surely does not mean that rate-aided hospitals are now to enter into competition with the Voluntary Hospitals in the sphere which the latter have successfully filled.

5. The said Voluntary Hospitals are most willing to co-operate fully and fairly with Local Authorities, but :—

(*a*) In some cases this could only be successfully and satisfactorily accomplished under the guidance and with the assistance of a neutral agency, preferably the Department of Health.

(*b*) Such co-operation could not be arranged satisfactorily on a national basis, as the problems, needs and circumstances of different parts of Scotland differ materially.

6. The said Voluntary Hospitals recognize the usefulness and importance of having some agency to speak and act for them. The British Hospitals Association, representing Voluntary Hospitals throughout England, Scotland and Wales, is a most useful and active organization, but it was early recognized that it could not effectively deal with local problems and needs. Accordingly, England was divided into various regions with a Sub-Committee in each. Scotland and Wales were regarded as two districts, the Scottish Committee being called the Scottish Branch of the British Hospitals Association. The Scottish Branch is a useful organization, but it is difficult to gather together delegates from all parts of the country except on special occasions, and it is impossible to interest, say, Inverness or Dundee with matters peculiarly applying, say, to Lanarkshire.

It was accordingly proposed that the Scottish Branch should have three Regional Committees—one for the West, one for the East, and one for the North. These Committees, unfortunately, have never functioned, but the representatives of the said Voluntary Hospitals in Glasgow and the West of Scotland who presented the above-mentioned Memorandum, and are members of the Scottish Branch of the British Hospitals Association, have now formed themselves into the Western Regional Committee of said Scottish Branch, in order that the Voluntary Hospitals in Glasgow and the West of Scotland may have a proper and fully representative organization to speak and act for them in the future, either in negotiations with the Department of Health or Local Authorities or otherwise. Sir James Macfarlane, Chairman, and Mr. Morrison Smith, Secretary, both of the Royal Infirmary, Glasgow, have been appointed Chairman and Secretary of the Western Regional Committee. It is as well to emphasize the fact that the negotiating body must be representative of and in touch with all the Voluntary Hospitals in the district, and not consist of two or three more or less self-appointed individuals, whatever their position may be.

7. The Western Regional Committee will be prepared, either directly or through an Executive Committee, if desired, to submit and discuss schemes for co-ordination of health services and co-operation between hospitals in their district, subject always to what is above written regarding the meaning of these terms co-ordination and co-operation.

8. If the Voluntary Hospitals are to be faced with undue competition on the part of the rate-aided hospitals, they will require to reconsider their position and re-arrange their sphere of operations.

9. In considering the question of co-ordination and co-operation, it is essential to keep carefully in view that all the large Voluntary Hospitals, and particularly those with teaching schools, are not Local Institutions, but are meeting the needs for hospital services over a wide area, *e.g.* The Royal, Western and Victoria Infirmaries, Glasgow, and the Royal

Hospital for Sick Children, Glasgow, are receiving patients from the extreme North to the Solway and from all the Western Isles. It would therefore be a mistake to act as if the Corporation of Glasgow was the only Local Authority interested in the work of these Institutions. The outlying districts in the West are to a large extent dependent on such Institutions for the necessary hospital services—hence the question of co-ordination is primarily a matter for the Department of Health.

10. It is essential to the continuance of the voluntary system :—

(*a*) That consistent with being a corporate part of the Health Services of the country the individuality, the independence, and the idea of being to a certain extent a local possession must be carefully preserved in the case of each hospital.

(*b*) That each hospital should be responsible for the management of its own affairs.

(*c*) That the Directors, Ladies' Committee, Almoners' Committee, Physicians, Surgeons, Nurses, Collectors and all the personnel that goes to make up a first-class Voluntary Hospital should have a feeling of *camaraderie* and *esprit de corps*, with a personal pride in and determination to ensure the progress of their own Institution. It is this somewhat indefinable element that makes the difference between a Voluntary Hospital and one supported by the rates.

(*d*) That, by whatever means the requisite co-ordination and co-operation may be accomplished, it must not result in the dictatorship either of an individual or a committee or supersede the authority or judgment of those in control of the hospital ; otherwise they will conclude that they are no longer required and will resign. All local influence would thus be lost.

(*e*) That when fresh burdens and duties are laid on Voluntary Hospitals as a result of statutory provisions or otherwise the cost to said hospitals should not be lost sight of in arranging for remuneration in the same connection in other directions.

(*f*) That the State should not regard Voluntary Hospitals only with what may be called a kind of benevolent neutrality, but should co-operate with and assist them in every way possible, financially and otherwise, recognizing that they are doing an immense amount of good and essential work and are an important part of the health services of the country. On the matter of finance it should be kept in view that in many countries and in many parts of England and a few districts in Scotland grants or payments are made by Local Authorities to Voluntary Hospitals in recognition of the medical and surgical work they are doing for the community and in relief of rates, and that such payments are made to help the Voluntary Hospitals and without any stipulations as to management or control on the part of Local Authorities.

